# Analysis of clinical aspects of leprosy patients between 2010–2017 at a reference center in Campinas^☆^^☆☆^

**DOI:** 10.1016/j.abd.2019.05.006

**Published:** 2020-02-12

**Authors:** Marcel Alex Soares dos Santos, José Luis Braga de Aquino, Elisangela Pegas, Elaine Cristina Faria Abrahao Machado

**Affiliations:** a Department of Dermatology, Faculdade São Leopoldo Mandic de Campinas, Campinas, SP, Brazil; b Department of Head, Neck and Chest Surgery, Pontifícia Universidade Católica de Campinas, Campinas, SP, Brazil; c Leprosy, Bullous and Phototherapy Clinic, Pontifícia Universidade Católica de Campinas, Campinas, SP, Brazil; d Dermatology Clinic, Pontifícia Universidade Católica de Campinas, Campinas, SP, Brazil

Dear Editor,

Leprosy is a contagious, chronic, granulomatous disease caused by the gram-positive bacillus Mycobacterium leprae.^1^

It mainly affects the skin and the peripheral nerves, but it can also compromise other organs. May have neurological and motor sequelae and have high disabling power if not treated early.^2^

It is still a serious public health problem and is culturally and socially linked to a stigmatizing condition. It is the main infectious cause of disability in man. In 2017, 210,671 new cases were detected in the world, Brazil being the 2nd country in the world with the highest prevalence of the disease. In 2017, the country recorded 26,875 new cases of leprosy, representing 12.7% of the global total of new occurrences.^2^, ^3^

Knowing the context of leprosy patients in a university hospital, with reference service in the treatment of the disease, contributes to the formulation of strategies in public health aimed at their control and gives subsidy to actions to face this local grievance.

This is an epidemiological, descriptive, retrospective study whose objective is to analyze the relationship of the clinical variables of leprosy in the patients reported in an outpatient dermatology clinic reference in leprosy in the city of Campinas in the period of 2010–2017, by analyzing the information contained in the medical records of these patients (*n* = 111).

The relationship between the variables was assessed using the Chi-Square test, Student's *t*-test or ANOVA followed by Tukey's multiple comparison test. The significance level considered was 5%.

In table 1 it is possible to see the relation of the operational classification with the other variables. Significant association of the presence of neural symptoms with operational classification (*p* < 0.0001) was observed. There are more patients in the multibacillary classification with neural symptoms (*p* < 0.0001), as well as physical incapacity (*p* = 0.0124).

**Table 1:** Comparison between operational classifications groups (Chi-square test).

Variable	Category	Operational classification				Total		*p*-Value
		MB		PB		*n*	%	
		*n*	%	*n*	%			
Gender	Female	37	43.5	10	38.5	47	42.3	0.6472
	Male	48	56.5	16	61.5	64	57.7	

Clinical form	Boderline	35	41.2	0	0.0	35	31.5	<0.0001
	Indeterminate	4	4.7	9	34.6	13	11.7	
	Pure neural	3	3.5	1	3.8	4	3.6	
	Tuberculoid	3	3.5	16	61.5	19	17.1	
	Lepromatous	40	47.1	0	0.0	40	36.0	

Neural symptoms	No	7	8.8	12	46.2	19	17.9	<0.0001
	Yes	73	91.3	14	53.8	87	82.1	

Physical disability	No	20	37.0	11	73.3	31	44.9	0.0124
	Yes	34	63.0	4	26.7	38	55.1	

In the analysis of the clinical form with the other variables, it was observed an association of the clinical form with presence of neural symptoms (*p* = 0.0002), patients with the virchowian form and dimorphic have more neural symptoms.

Table 2 shows the comparison of the age of diagnosis and the other variables studied. There was a significant difference in the age of diagnosis in relation to the operational classification (*p* = 0.0001), patients with paucibacillary classification had a lower age of diagnosis. Patients with clinical virchowian form (*p* = 0.0112), those with neural symptoms (*p* = 0.0278) and with physical disability (*p* = 0.0102) present a higher age of diagnosis.

**Table 2:** Comparison of the age of diagnosis among the variables.

Variable	Category	*n*	Average	SD	Minimum	Median	Maximum	*p*-Value
Operational classification	Multibacillary	85	50.3	16.4	15.0	51.0	87.0	0.0001
	Paucibacillary	26	35.7	16.1	8.0	29.5	74.0	

Clinical form	Boderline	35	50.5	14.7	21.0	49.0	81.0	0.0112
	Indeterminate	13	33.5	15.3	10.0	27.0	61.0	
	Pure neural	4	41.5	34.9	8.0	41.5	75.0	
	Tuberculoid	19	42.7	14.3	25.0	42.0	74.0	
	Lepromatous	40	50.6	17.5	15.0	51.5	87.0	

Neural symptoms	No	19	38.3	13.6	10.0	42.0	61.0	0.0278
	Yes	87	47.7	17.2	8.0	48.0	81.0	

Physical disability	No	31	40.3	16.5	8.0	40.0	70.0	0.0102
	Yes	38	50.4	15.2	18.0	51.0	79.0	

Baciloscopy pre treatment	Negative	72	46.7	17.6	8.0	45.5	81.0	0.8392
	Positive	39	47.4	17.1	15.0	48.0	87.0	

Baciloscopy after treatment	Negative	83	46.8	16.5	8.0	48.0	78.0	0.4167
	Positive	9	42.1	16.4	27.0	38.0	79.0	

Table 3 shows the associations of neural symptoms with the other variables. Patients with neural symptoms have more physical disability (*p* = 0.0159), have more positive bacilloscopy before treatment (*p* = 0.0139), use more frequent PQT-MB therapeutic scheme (*p* < 0.0001) and extend the treatment more than the predetermined time (*p* = 0.0172).

**Table 3:** Comparison between groups of neural symptoms (Chi-square test).

Variable	Category	Neural symptoms				Total		*p*-Value
		No				*n*	%	
		*n*	%	*n*	%			
Gender	Female	10	52.6	34	39.1	44	41.5	0.2775
	Male	9	47.4	53	60.9	62	58.5	

Physical disability	No	8	80.0	23	39.0	31	44.9	0.0159
	Yes	2	20.0	36	61.0	38	55.1	

Baciloscopy pretreatment	Negative	17	89.5	52	59.8	69	65.1	0.0139
	Positive	2	10.5	35	40.2	37	34.9	

Baciloscopy after treatment	Negative	17	100.0	64	87.7	81	90.0	0.1270
	Positive	0	0.0	9	12.3	9	10.0	

Treatment	PQT-MB	7	36.8	74	85.1	81	76.4	<0.0001
	PQT-PB	12	63.2	13	14.9	25	23.6	

Extended treatment	No	16	94.1	54	65.1	70	70.0	0.0172
	Yes	1	5.9	29	34.9	30	30.0	

In relation to the presence of physical disability, it was observed that male patients have more physical disability (*p*-value = 0.0273), as well as patients with multibacillary treatment regimen (*p*-value = 0.0046).

Multibacillary patients are twice as likely to have physical disabilities as paucibacillary patients because multibacillary patients have a high bacillary load in the body and because they have a generally more clinical picture, the bacilli act longer on the nerves, causing neural symptoms and leading to the disabilities.^4^

The virchowian clinical form has the greatest impact on the development of physical disabilities and deformities, increasing the chance of developing Grade II disability in 16.5 times.^2^

Patients older than 60 years are twice as likely to be multibacillary as young people are less exposed to *Mycobacterium leprae* and the development of the multibacillary form requires a long incubation period of the bacillus. Elderly patients present age-related immunological changes and increased susceptibility to infections with monocytes and neutrophils with impairment of functions, reduction of phagocytic capacity, decreased antigen presentation and change in the cytokine profile of Th1 to Th2.^5^

Men are 1.4 times more likely to develop physical disabilities than women because they take less health care, delaying the diagnosis and treatment of the disease. With more time of the bacillus acting actively in the organism, the greater the neural damage.^2^, ^5^

The study presented some limitations. Although the dermatology outpatient clinic of the study met a wide demand in the city of Campinas-SP, only the patients notified at the site were included in the study. A large percentage of patients were not evaluated for the presence of physical disabilities (37.8%). The study was based on the classification of Madrid and the operational one presented by the WHO. It is worth noting that, unlike Ridley and Jopling's classification, the classifications used do not separate the dimorphic patients into DT, DD and DV, which present clinical, bacteriological and immunological differences.

## Financial support

None declared.

## Authors’ contributions

Marcel Alex Soares dos Santos: Statistic analysis; approval of the final version of the manuscript; conception and planning of the study; elaboration and writing of the manuscript; obtaining, analysis, and interpretation of the data; critical review of the literature; critical review of the manuscript.

José Luis Braga de Aquino: Approval of the final version of the manuscript; conception and planning of the study; effective participation in research orientation.

Elisangela Pegas: Conception and planning of the study; intellectual participation in the propaedeutic and/or therapeutic conduct of the studied cases.

Elaine Cristina Faria Abrahao Machado: Conception and planning of the study; elaboration and writing of the manuscript.

## Conflicts of interest

None declared.
